# Phylogeographic and genetic characterization of porcine circovirus type 2 in Taiwan from 2001–2017

**DOI:** 10.1038/s41598-019-47209-1

**Published:** 2019-07-25

**Authors:** Guang-Ting Tsai, Yung-Cheng Lin, Wei-Hao Lin, Jih-Hui Lin, Ming-Tang Chiou, Hsin-Fu Liu, Chao-Nan Lin

**Affiliations:** 1 Department of Veterinary Medicine, College of Veterinary Medicine, National Pingtung University of Science and Technology, Neipu, Pingtung Taiwan; 2 Animal Disease Diagnostic Center, National Pingtung University of Science and Technology, Neipu, Pingtung Taiwan; 3 Department of Medical Research, Mackay Memorial Hospital, Taipei, Taiwan; 4 Department of Nursing, Shu-Zen junior College of Medicine and Management, Kaohsiung, Taiwan; 5 Center of Diagnostics and Vaccine Development, Centers for Disease Control, Taiwan, Taipei Taiwan; 6 Research Center for Animal Biologics, National Pingtung University of Science and Technology, Pingtung, Taiwan; 7 Department of Bioscience and Biotechnology, National Taiwan Ocean University, Keelung, Taiwan; 8 Department of Nursing, National Taipei University of Nursing and Health Sciences, Taipei, Taiwan

**Keywords:** Evolutionary biology, Viral evolution

## Abstract

Porcine circovirus type 2 (PCV2) is an important pathogen that causes significant economic losses in the swine industry worldwide. Five major PCV2 genotypes have been identified, including PCV2a, PCV2b, PCV2c, PCV2d, and PCV2e. To investigate the prevalence and phylodynamics of the different PCV2 genotypes in Taiwan, 214 PCV2 ORF2 sequences from Taiwan and other countries were analyzed. Genotypic differences were observed among PCV2a, 2b, and 2d at amino acid position 89 in ORF2, with isoleucine (I), arginine (R), and leucine (L), respectively. Similar to other countries, a genotypic shift was also observed in Taiwan, where the predominant genotype shifted from PCV2b to 2d after 2010. The estimated nucleotide substitution rate of Taiwanese strains in the ORF2 region was 8.467 × 10^−4^ substitutions per site per year. This rapid evolution rate of PCV2 may lead to the genotypic shift observed in Taiwan. The times to the most recent common ancestor (TMRCA) for PCV2a, -2b, and -2d-2 was dated to 1970, 1992 and 2004, respectively. Thus, the PCV2a, -2b, and -2d genotypes were already present in Taiwan before the introduction of the PCV2 vaccine.

## Introduction

Porcine circovirus type 2 (PCV2) is the causative agent of porcine circovirus-associated diseases (PCVADs)^1^, which causes enormous economic losses in the swine industry worldwide. Based on clinical symptoms and laboratory diagnosis, a number of diseases have been associated with PCVADs, including subclinical infection, systemic disease or postweaning multisystemic wasting syndrome (PMWS), lung disease, enteric disease, reproductive disease, porcine dermatitis and nephropathy syndrome (PDNS), and congenital tremors^2^. This disease has multifactorial syndromes and factors that are currently thought to influence the outcome of PCV2 infection, including the strains, host, coinfection with other pathogens, and immune modulation^1^.

PCV2 belongs to the genus *Circovirus* and the family *Circoviridae*^3^. PCV2 is a small, non-enveloped, circular, single-stranded DNA virus consisting of a circular genome of 1767–1768 nucleotides^3^, with at least four open reading frames (ORFs). ORF1 encodes two proteins associated with replication, designated Rep and Rep’^4,5^. ORF2 encodes the capsid protein^6^, which interacts with the host receptor and induces immune responses and is a suitable marker for phylogenetic and epidemiological analysis of PCV2^7,8^. ORF3 encodes a protein that is thought to play a role in apoptosis^9^, while ORF4 encodes a newly discovered protein with a role in suppressing caspase activity and regulating CD4+ and CD8+ T lymphocytes^10^. However, several researchers have noted that the existence of ORF3 and ORF4 is dubious due to lack of reliable experiments in pigs and poor reproducibility^11,12^. For PCV2 pathogenesis of T lymphocyte depletion, PCV2 has been demonstrated to have the ability to impact T-cell selection processes in the thymus and then impair thymocyte maturation^13^.

Currently, PCV2 strains are further classified into five genotypes (PCV2a, PCV2b, PCV2c, PCV2d, and PCV2e) based on ORF2 classification criteria^8,14–16^. PCV2a was the prevalent PCV2 genotype in the global pig population until 2000^8^, whereas PCV2b has existed in Europe and Asia since 1997^17^. Since PCV2b appeared in North America in 2005, it has been the predominant genotype in the United States pig population^16,18^. PCV2c was identified in a retrospective investigation of porcine serum samples from 1980, 1987, and 1990 in Denmark^17^ and was recently detected in a feral pig in Brazil^19^. PCV2d can be subclassified into PCV2d-1 and PCV2d-2^15^. The PCV2d-1 genotype was first identified in 2002 in China^20,21^, whereas the PCV2d-2 genotype was first recognized in 2008 in China, with both genotypes having been linked with increased virulence^15,22,23^. Until 2016, PCV2d-2 was the predominant genotype in the United States^16^, and PCV2e has been present in the United States since at least 2006, as determined by a retrospective investigation^14^. Due to an extra 12 nucleotides present at the 3′ end of ORF2, PCV2e was thought to be a progenitor of PCV2a-d^14^.

**Figure 1:**
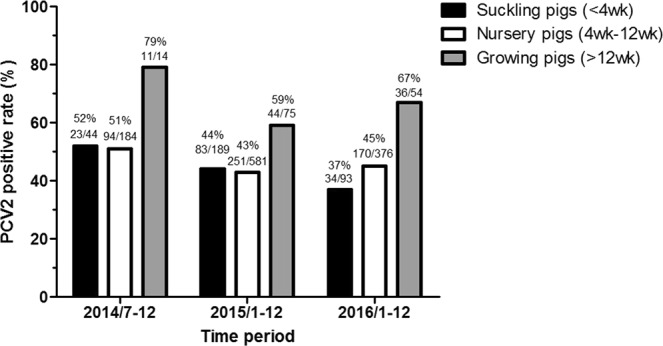
PCV2 positivity rate in swine samples deposited at the ADDC of NPUST in Taiwan from July 2014 to December 2016. The numbers above bars indicate the number of PCV2-positive pigs out of the total number of pigs in which detection was attempted.

In Taiwan, the first PCV2 vaccine was introduced in September 2010. PCV2 vaccination is effective, inducing neutralizing antibodies and significantly reducing PCV2-associated lesions and PCV loads in pigs^24^. The positive rates of the PCV2 antigen in suckling, nursery and growing pig herds was determined to be 36.6–52.3%, 43.2–51.1%, and 58.7–78.6%, respectively, based on the database from the Animal Disease Diagnosis Center of National Pingtung University of Science and Technology (ADDC of NPUST) from 2014 to 2016 in Taiwan (Fig. 1). Thus, it appears that PCV2 still plays an important role in the pig industry in Taiwan. Although newly emerging strains and global genotype shifts have been continually reported, little is known regarding the genetic diversity of PCV2 in Taiwan^25,26^. To better understand the evolutionary history of PCV2 in Taiwan, in this study, we conducted a comprehensive phylodynamic and phylogeographic analysis based on the complete ORF2 gene sequences from Taiwanese PCV2 strains.

## Results

### Specimen selection and histopathological examination

**Figure 2:**
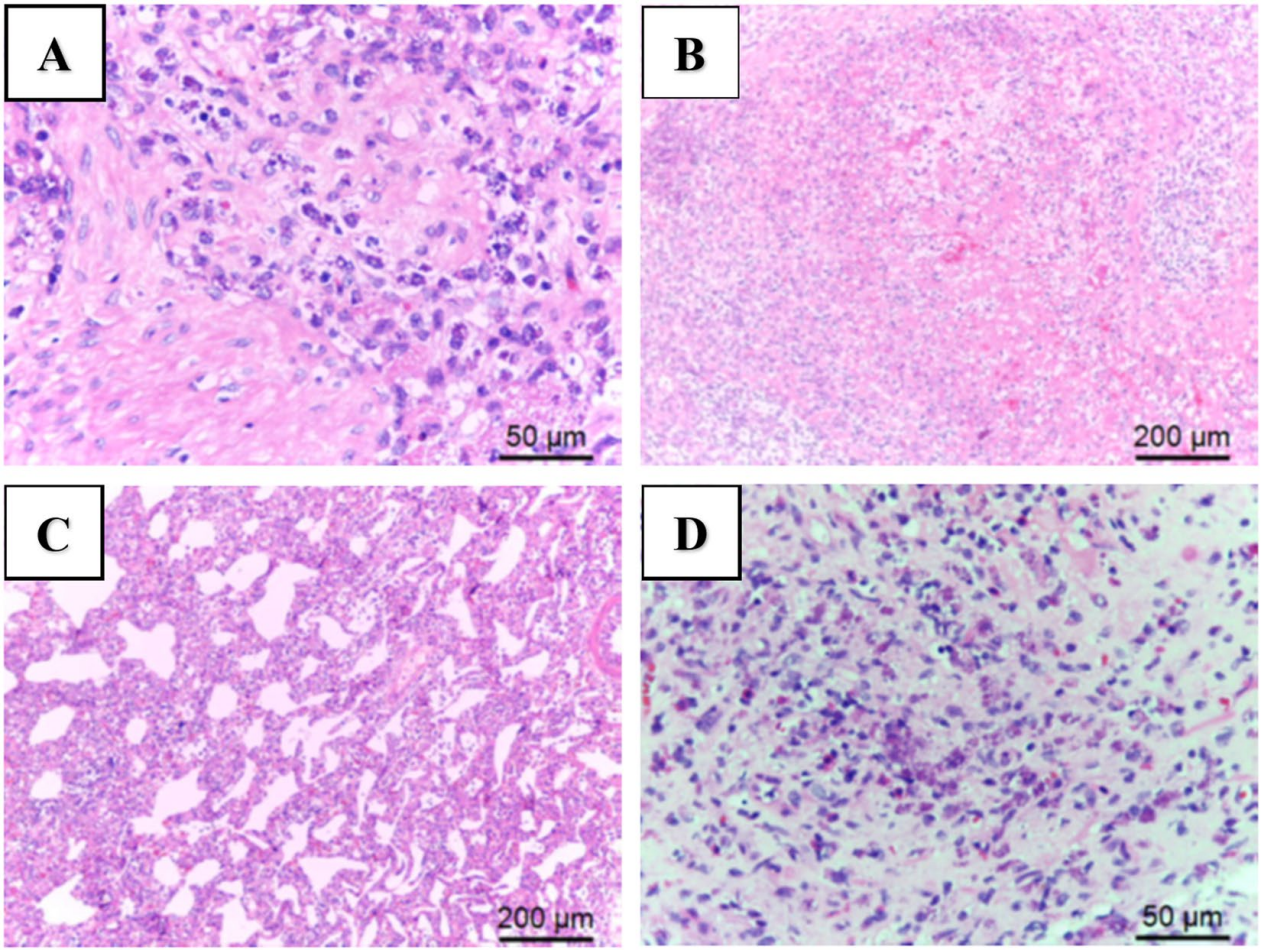
Detection of histopathological lesions in typical PCVAD clinical cases by hematoxylin-eosin staining. (**A**,**B**) Necrotizing lesion of the submandibular lymph node with basophilic endoplasmic inclusion bodies in mononuclear cells. (**C**) The alveolar septa were thickened by interstitial infiltration of mononuclear inflammatory cells. (**D**) The mesenteric lymph node contained basophilic endoplasmic inclusion bodies in mononuclear cells.

**Figure 3:**
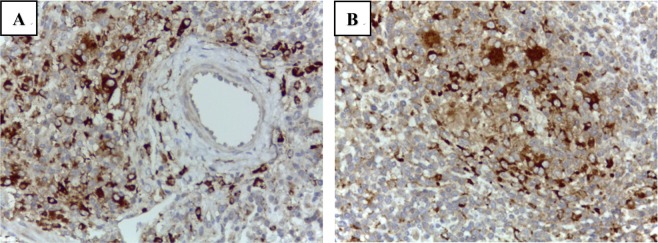
PCV2 immunochemistry of typical PCVAD clinical cases. (**A**) The interstitial pneumonia lesion contained PCV2 antigen positive signals (brown) within the cytoplasm of macrophage-like cells. (**B**) PCV2 immunolabeling (brownish signal) is seen in the cytoplasm of macrophage-like cells in the lymph node.

Sixty specimens (42 necropsied pigs and 18 serum samples) were selected from 1,092 necropsied pigs and 724 serum samples from the ADDC of NPUST from January 2016 to February 2017. All cases were diagnosed as PCVAD based on the PCV2 load being higher than 10^7^ DNA copies/ml in serum and homogenized tissues^1,27^. Histopathological examinations were performed from 42 specimens, the results of which showed that mononuclear cells infiltrated the alveolar septa, with necrotizing submandibular or mesenteric lymph nodes that contained basophilic endoplasmic inclusion bodies (Fig. 2). The PCV2 immunochemistry staining results for typical PCVAD cases showed a PCV2 antigen positive signal (brown) located within the cytoplasm of macrophage-like cells in alveolar septa and lymph nodes (Fig. 3).

### Genotype prevalence of PCV2 from 2001 to 2017 in Taiwan

**Figure 4:**
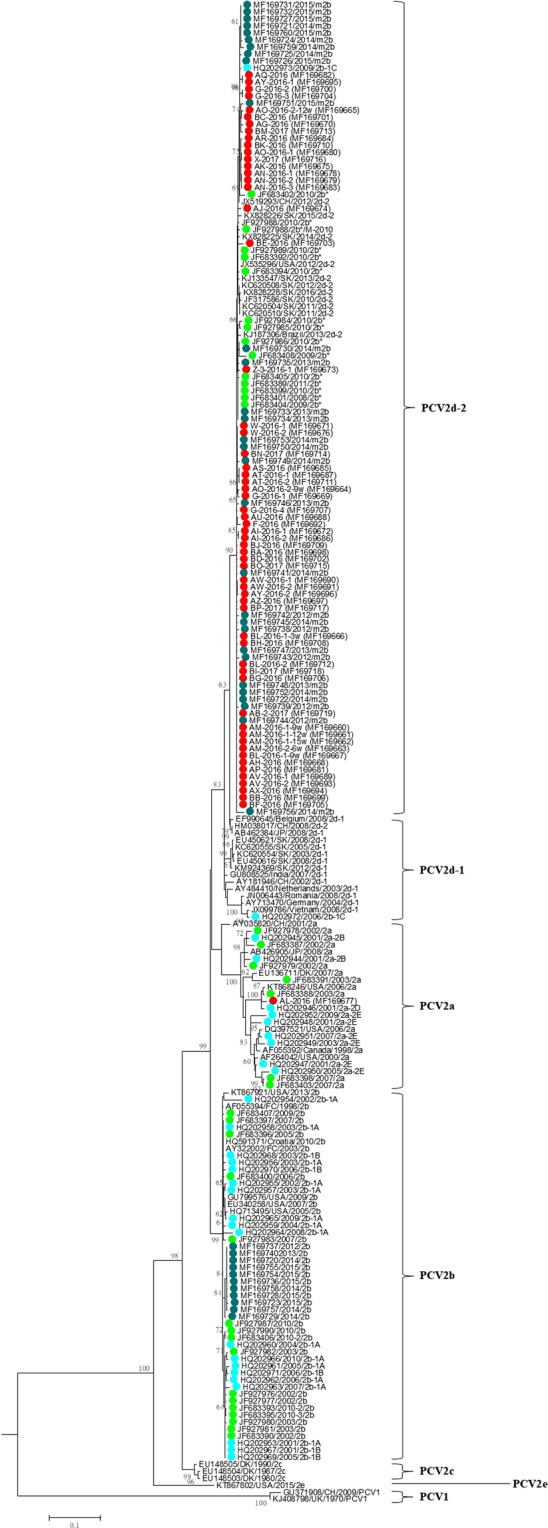
Phylogenetic tree based on PCV2 ORF2 gene sequences. The tree was constructed using the maximum-likelihood method based on the general time reversible (GTR) model with a gamma-distributed rate (five rate categories) and an invariant site (I). Taiwanese PCV2 strains were labeled with solid dots of different colors: red, blue-green, bright-green, and bright-blue dots represent samples collected from 2016–2017, 2012–2015, 2002–2011, and 2001–2010, respectively.

Two hundred fourteen PCV2 ORF2 sequences were used to construct a maximum-likelihood (ML) tree, including 168 sequences from Taiwan and 46 reference sequences from the NCBI database (Table 1). The ML tree indicated that the predominant PCV2 genotype was different at different collection times. Our ORF2 sequence data revealed that both the PCV2a and PCV2b genotypes were prevalent PCV2 field strains circulating in Taiwan before 2007 (Table 2). After 2010, the predominant genotype shifted from PCV2b to 2d until today (Fig. 4) (Table 2), with PCV2a existing as a minor strain in Taiwan in the last decade (Table 2).

**Table 1:** The details of PCV2 sequences used in this study.

Accession number (total strains)	Time period (year)	Country
HQ202944-HQ202973	2001–2010	Taiwan
JF683387-JF683408, JF927976-JF927990	2002–2011	Taiwan
MF169720-MF169760	2012–2015	Taiwan
MF169660-MF169719	2016–2017	Taiwan
AB426905, AY322002, EU136711, HQ591371	2003–2010	Croatia, Denmark, France, Japan
AB462384, AF055392, AF055394, AF264042, AY035820, AY181946, AY484410, AY713470, DQ397521, EF990645, EU148503, EU148504, EU148505, EU340258, GU799576, GU808525, HM038017, HQ713495, JN006443, JX099786, JX519293, JX535296, KJ187306	1980–2013	Belgium, Brazil, Canada, China, Denmark, France, Germany, India, Japan, Netherlands, Romania, USA, Vietnam
EU450616, EU450621, GU371908, JF317586, KC620504, KC620508, KC620510, KC620554, KC620555, KJ133547, KJ408798, KM924369, KT867802, KT867921, KT868246, KX828224, KX828225, KX828226, KX828228	1970–2016	China, UK, USA, South Korea

**Table 2:** Genotypic analysis of 168 Taiwanese PCV2 sequences collected from 2001 to 2017.

Year	Genotype				
	PCV2a	PCV2b	PCV2d-1	PCV2d-2	Total
2001	5	2	0	0	7
2002	3	5	0	0	8
2003	3	7	0	0	10
2004	0	2	0	0	2
2005	1	3	0	0	4
2006	0	4	1	0	5
2007	3	3	0	0	6
2008	0	1	0	1	2
2009	1	2	0	3	6
2010	0	6	0	10	16
2011	0	0	0	1	1
2012	0	1	0	6	7
2013	0	1	0	6	7
2014	0	4	0	12	16
2015	0	5	0	6	11
2016	1	0	0	52	53
2017	0	0	0	7	7
Total	17	46	1	104	168

### Evolution rate, phylodynamics, and phylogeography of PCV2

**Figure 5:**
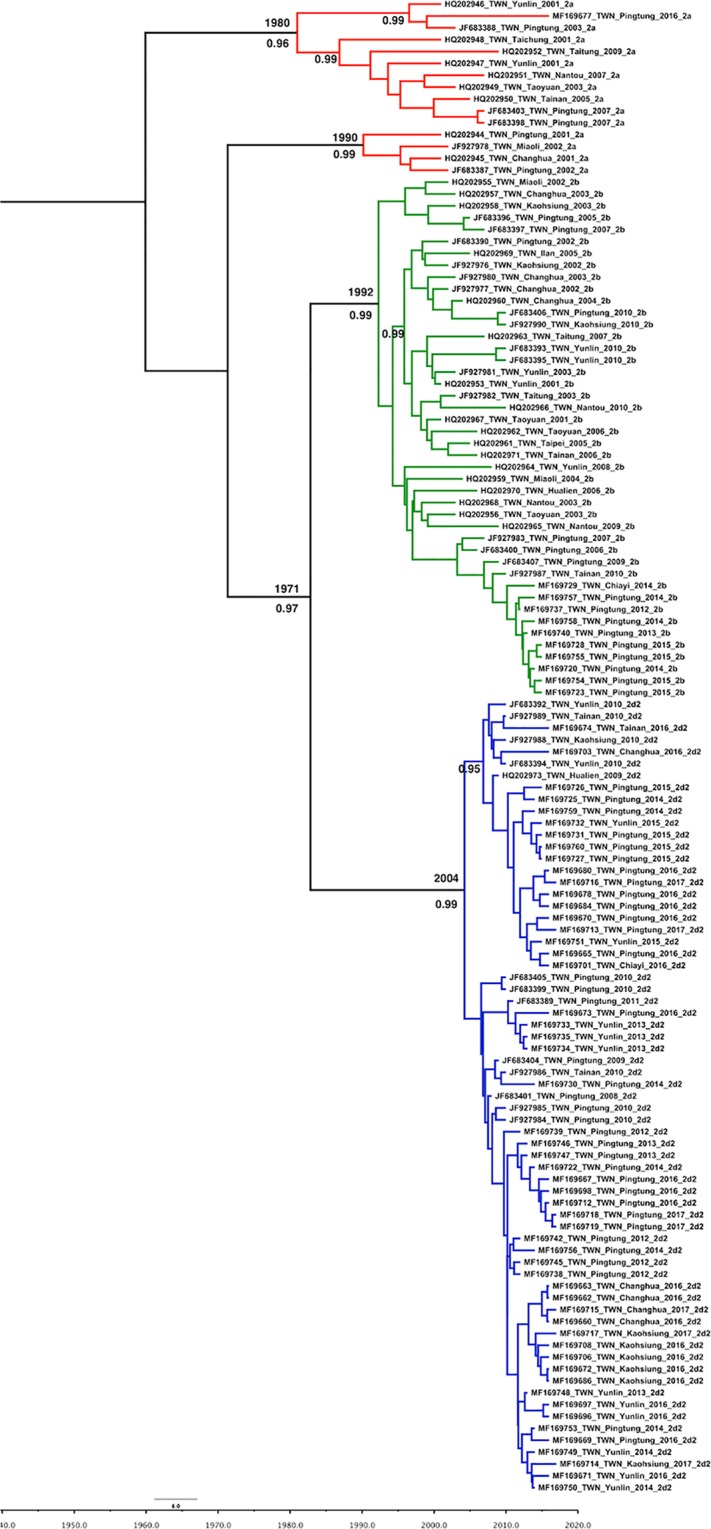
Maximum-clade-credibility (MCC) tree of PCV2 inferred from 126 complete ORF2 sequences from Taiwan. The MCC tree was constructed with a 10% burn-in using Tree Annotator v 1.10 implemented in the BEAST software package. The red, green and blue branch lines represent the PCV2a, PCV2b, and PCV2d isolates, respectively. The numbers adjacent to the branches are the posterior probability values and branch times. Only posterior probability values greater than 0.95 are shown.

One hundred twenty-six Taiwanese sequences with associated collection time and location data were included in the phylodynamic analysis. The uncorrelated exponential relaxed clock model and Bayesian skyline were determined as the best fit models. The estimated nucleotide substitution rates of the PCV2 ORF2 region were 8.467 × 10^−4^ substitutions per site per year (the 95% highest posterior density (HPD) was 5.973 × 10^−4^ to 1.132 × 10^−3^). The time to the most recent common ancestor (TMRCA) of PCV2 was estimated to be 1957 (95% HPD: 1926–1982), while for the PCV2d-2 group, the TMRCA was dated to 2004 (95% HPD: 1999–2007) among Taiwanese sequences. The evolutionary rate of the synonymous positions was 1.664, twice that of the nonsynonymous positions (0.668), in the ORF 2 region (Table 3). The Taiwanese PCV2 strain diverged in approximately 1980 and 1990 for PCV2a, 1992 for PCV2b, and 2004 for the PCV 2d-2 lineage (Fig. 5).

**Table 3:** Mean relative evolutionary rates for codon positions and TMRCA in the ORF2 gene region of Taiwanese strains.

	TMRCA	Substitution Rates Sub/Site/Year (10^−4^)	Mean Relative Substitution Rate	SE of Mean
ORF2 region		8.467 (5.973–11.326)^a^		
PCV2	1957 (1926–1982)^a^			
PCV2a Taiwanese strains	1970 (1947–1987)^a^			
PCV2b Taiwanese strains	1992 (1985–1998)^a^			
PCV2d-2 Taiwanese strains	2004 (1999–2007)^a^			
1st + 2nd codon position			0.668 (0.568–0.769)^a^	5.405E-4
3rd codon position			1.664 (1.462–1.864)^a^	1.081E-3

^a^() Lower and upper 95% of highest posterior density (HPD).

**Figure 6:**
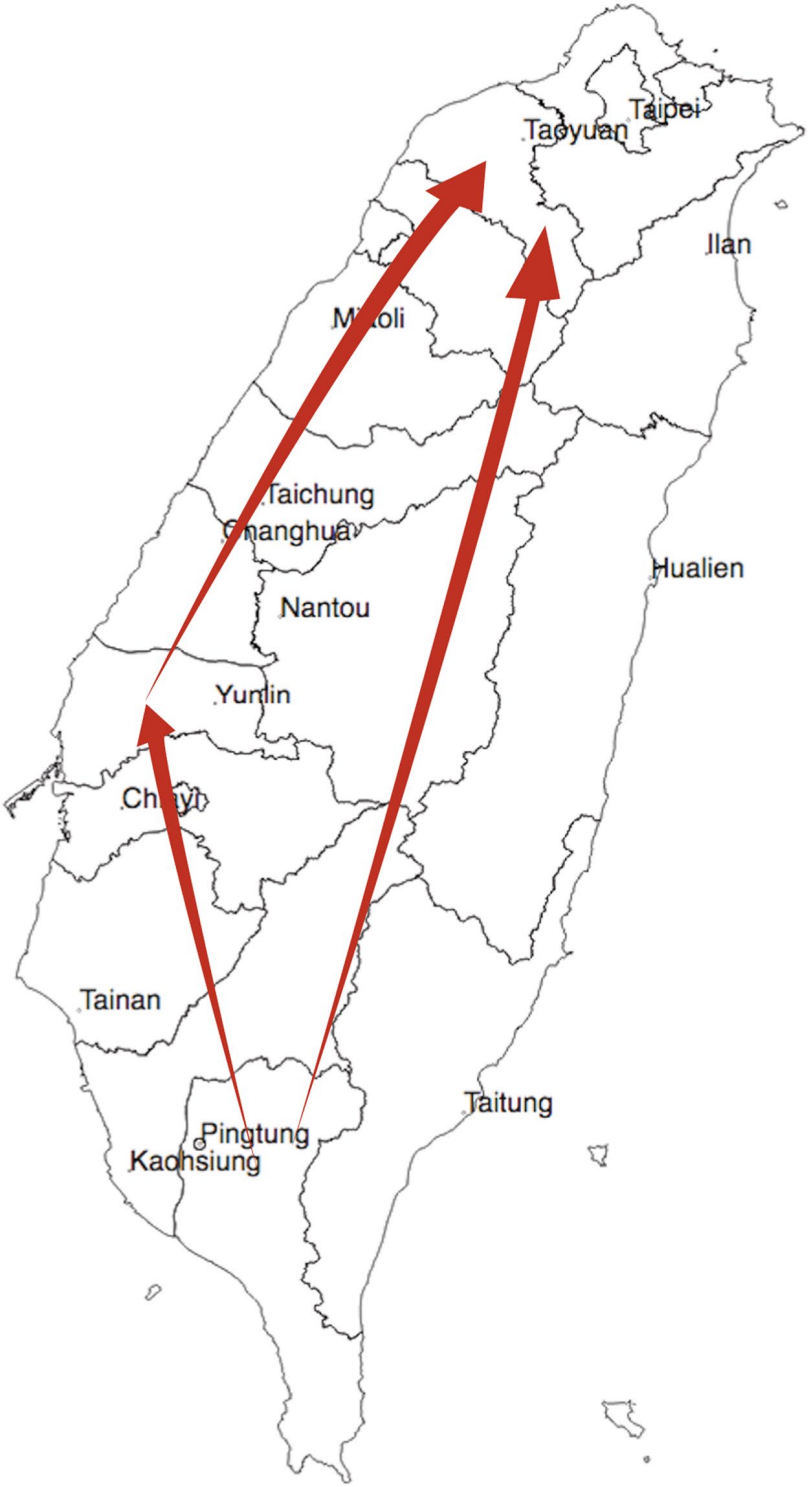
Migration of temporal dynamics for PCV2 in Taiwan. The visualizing location annotated Maximum Clade Credibility tree by SPREAD3 software. The branches show an overview of the possible transmission routes of PCV2 in Taiwan. The direction of the spread of the virus is indicated with arrows.

**Figure 7:**
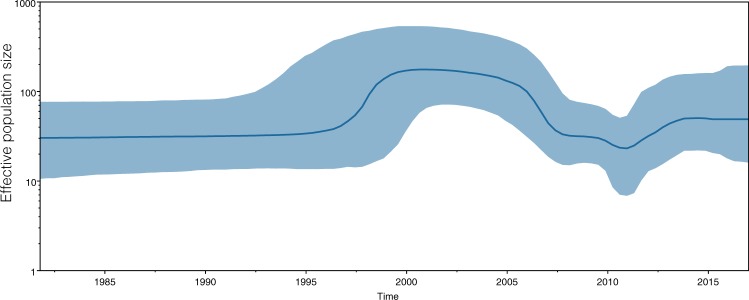
Bayesian skyline plot for the complete capsid gene encoding PCV2. The x-axis shows the years before 2017, while the y-axis represents effective viral population size. The thicker bold line represents the median estimate of the effective number of infections over time, whereas the thinner blue lines indicate the upper and lower bounds of the 95% HPD.

The results of phylogeographic analysis indicated the possible transmission route of PCV2 in Taiwan’s pig industry. The earliest source of the virus was in southern Taiwan (Pingtung County), and it subsequently spread to central Taiwan (Yunlin and Changhua Counties) and northern Taiwan (Taoyuan). Finally, the virus disseminated to all regions of the country (Fig. 6). The phylodynamics of PCV2 was estimated by a Bayesian skyline plot (BSP) based on the capsid protein gene. The effective population size of PCV2 was stable until 1997, after which it increased significantly until 2000 and subsequently exhibited a significant decrease until 2011 (Fig. 7). After 2011, the effective population size slowly returned to stability.

### Nucleotide sequence comparison and amino acid sequence analysis of the PCV2 capsid protein region

Different lengths of the PCV2 ORF2 region were detected in the Taiwanese isolates (702 and 705 nucleotides; nt), which was due to the insertion AAG or AAA (lysine) at position 700 at the 3′ end of the capsid protein gene of PCV2d-2 from Taiwan. The nucleotide distance and identical amino acids within each genotype in Taiwan were 92–100% and 90.2–100%, respectively.

**Figure 8:**
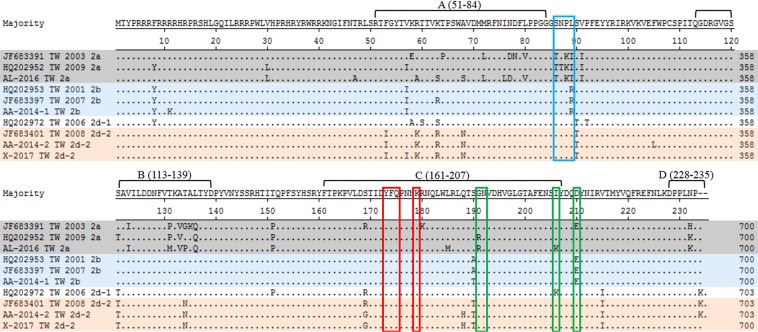
Amino acids of the putative PCV2 capsid protein for PCV2a (gray), PCV2b (blue), and PCV2d-1 and PCV2d-2 (orange). The four general antibody recognition regions are labeled (**A**) (51–84), (**B**) (113–139), (**C**) (161–207), and (**D**) (228–233). Positions 86–89 (blue box) were previously proposed to distinguish between PCV2a and PCV2b^29^. Positions 190–191, 206, and 210 (green box) are important for PCV2 replication *in vitro*^59^. Positions 173–175 and 179 (red box) are important for antibody recognition.

**Figure 9:**
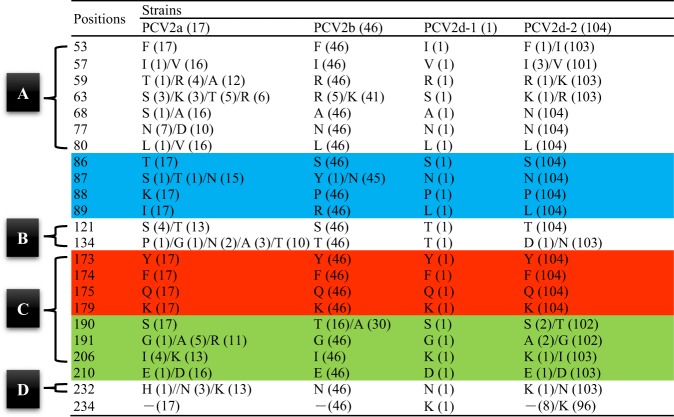
Variable residues in the putative capsid protein of ORF2 among 168 PCV2 isolates from Taiwan. Amino acid respective number was represented for amount of strains. Four general antibody recognition regions were labeled (**A**) (51–84), (**B**) (113–139), (**C**) (161–207), and (**D**) (228–233). Positions 86–89 (blue) were proposed to distinguish between PCV2a and PCV2b. Positions 173–175 and 179 (red) are important for antibody recognition. Positions 190–191, 206, and 210 (green) are important for PCV2 replication *in vitro*.

The commercial PCV2 vaccine was manufactured based on the PCV2a strain. To understand the protection ability of the PCV2a-based vaccine against toward PCV2d, several known antibody recognition sites were compared and classified into four regions in the schematic amino acid sequence of the putative PCV2 capsid protein. Regions A, B, C, and D represent positions 51–84, 113–139, 161–207, and 228–233, in the putative PCV2 capsid protein, respectively^7,28^, including those of PCV2a, PCV2b, PCV2d-1, and PCV2d-2 (Fig. 8). Several amino acid substitution sites were observed within the antibody recognition region between PCV2a and PCV2d-2, including F53I, A59K, A68N, V80L, T134N, R191G, K206I, K232N, and 234K. Furthermore, position 89, which is proposed to distinguish between PCV2a and PCV2b^29^, was the primary substitution site between genotypes. Position 89 was isoleucine in PCV2a, arginine in PCV2b, and leucine in PCV2d. Interestingly, the important antibody recognition residues 173–175 and 179^30^ were conserved in each genotype (Fig. 9).

## Discussion

PCV2 is an important pathogen in most pig-producing countries, with an observed prevalence of PCV2 in Taiwan of 92% in herds and 68.8% in pigs. PCV2 is also responsible for 90.4% of cases in porcine respiratory disease complex (PRDC) in Taiwan^31,32^. These data reveal that PCV2 infection is severe in the field, demonstrating that strategies to control the situation must be identified. The clinical symptoms of PCV2-infected pigs have improved significantly since PCV2 vaccine became available in Taiwan in September 2010. However, some sporadic PCVAD cases in pig farms were observed in our surveillance effort from 2014 to 2016. This situation was similar to the previous reports from the United States^16^. Reports from the United State and Korea also indicated that sporadic deadly cases of PCVAD occurred even if the pigs were vaccinated^33,34^. Furthermore, the genotyping results indicated that PCV2 clustered with a new genotype, PCV2b, which was defined as mutant PCV2b. Isolates from these cases were further sequenced and analyzed with respect to the ORF2 gene region and were subsequently defined as PCV2d-2 (previously known as mutant PCV2b)^15^.

Two genotypic shifts in PCV2 occurred during the 20th century, including PCV2a being replaced by PCV2b in 2005 and PCV2b shifting to PCV2d in 2008^15,35–37^. Since then, PCV2d has become a major genotype responsible for great losses in the swine industry worldwide, including the United States, South America, Europe, China, Korea, and Thailand^16,36,38^. The genotypic shift was observed in Taiwan too. PCV2b was the predominant genotype before 2007, but it was replaced by PCV2d-2 in 2010 in the swine farms of Taiwan^25^, and the latter continues to dominate. Indeed, the genotypic shift was observed in previous studies.

The primary commercial vaccines are manufactured from the PCV2a genotype or its capsid protein and came to market in 2006^1,39^. It is surprising that the first genotypic shift slightly predated the widespread vaccination against PCV2^36^. Whether the genotypic shift is due to the selective pressure by the PCV2 vaccine remains a disturbing issue in the search for a pig disease prevention strategy. A previous report indicated that the implementation of the PCV2a-based vaccine dramatically reduced PCV2a infection in the United States pig population, but vaccinated herds still had a 9.9% prevalence of PCV2b. This result suggested that accumulation of genetic variability could arise from selective pressures promoted by vaccination-induced immunity escape^39^. Genotypes that previously went uncirculated in a swine populations may therefore rapidly replicate better than the most prevalent genotype to which the swine population is highly protected^35^. Franzo *et al*. reported that the introduction of PCV2a-based vaccine markedly changes the evolution pattern of PCV2a but not that of PCV2b^40^. These results are consistent with those obtained in our study, as the observed nucleotides identity among PCV2a strains was 92–99.9%, which was more variable than that observed for PCV2b (95.2–100%) and PCV2d (97.3–100%). All of these results suggested that vaccination can effectively decrease PCV2a prevalence in herds, and the genetic variation of PCV2a has been facilitated by vaccine-induced selective pressure. This hypothesis is also supported by the BSP analysis in our study, which showed that the PCV2 population was stable for a long time and indicating that the gene variation of the PCV2 is steady in Taiwan. Strikingly, the PCV2 population appeared to slowly increase in approximately 2011 (Fig. 7). This phenomenon is in accordance with the time of PCV2 vaccination in Taiwan, suggesting that the PCV2a-based vaccine may introduce selection pressure on the virus that lead to a genotypic shift from PCV2a to PCV2d-2 in Taiwan.

The PCV2 ORF2 region has a high phylogenetic signal over the PCV2 genome. Therefore, the phylogenetic analysis based on the ORF2 of PCV2 is believed to be representative of a complete genome analysis^8,15^. Two genotypes, PCV2b-1C and the novel PCV2b, which were previously considered newly emerged genotypes of PCV2 in Taiwan^25,26^, were redefined in this study as PCV2d-1 or PCV2d-2 through phylogenetic analysis of ORF2 (Fig. 4). The time of the first isolated PCV2b, PCV2d-1, and PCV2d-2 strains in Taiwan could be traced back to in 2001, 2006, and 2008, respectively (Table 2). TMRCA was dated to 1970, 1992, and 2004 in Taiwanese strains of PCV2a, PCV2b, and PCV2d-2, respectively (Table 3), indicating that these genotypes had existed in Taiwan before the introduction of the PCV2 vaccine. A similar result was reported by Franzo *et al*., who observed that the TMRCA of PCV2a and PCV2b was from 1964 and 1973, respectively^35^. Furthermore, similar conclusions were drawn from the results of studies by Xiao *et al*.^15^ and Firth *et al*.^41^. Interestingly, the TMRCA of PCV2d was dated to 1958 in a study by Franzo *et al*.^35^, which was significantly earlier (46 years) than our results and those of Xiao *et al*. (i.e. year 1993)^15^ and suggests that Taiwanese PCV2d-2 is a young population. In addition, the PCV2 sequences used in those studies were downloaded from GenBank^15,35,41^, we analyzed the genotypes of local PCV2 sequences collected over approximately two decades. The substitution rates of the Taiwanese PCV2 lineage, which was estimated in our results to be 8.467 × 10^−4^ substitutions per site per year, was slightly lower than that observed by Xiao *et al*. (2.93 × 10^−3^ substitutions per site per year)^15^, Firth *et al*. (1.44 × 10^−3^ substitutions per site per year) and Franzo *et al*. (between 6.22 × 10^−4^–1.59 × 10^−3^ substitutions per site per year)^35^. Nevertheless, for a DNA virus, PCV2 did have a high substitution rate compared to the other RNA viruses^42^.

Phylogeographic analysis showed that the transmission route of PCV2 in Taiwan’s pig population likely originated from Pingtung County (southern Taiwan) then spread to Yunlin and Changhua Counties (central Taiwan) and finally to the entire island (Fig. 6). This transmission route appears to be reasonable, because most pig farms are in western Taiwan, especially in Pingtung, Yunlin, and Changhua Counties. The spread of the virus may be caused by the loose control of animal movement early on. Fortunately, strict animal transport and disease control procedures have been established in Taiwan. The results provided by our study will hopefully be used to increase the effectiveness of disease prevention.

Indeed, there is evidence for an impact of PCV2 vaccination on selection of the PCV2 sequence, especially on nucleotides involved in the formation of immune epitopes^37^, as we identified several amino acids substitutions in the capsid protein from different genotypes. The most interesting region was residues 86–89, which are proposed to distinguish between PCV2a (TNKI) and PCV2b (SNPR)^29^. However, we could easily differentiate PCV2a, PCV2b, and PCV2d by position 89, which harbored isoleucine (I), arginine (R), and leucine (L) residues, respectively (Table 3). The PCV2 capsid protein has the highest number of codons located in immune epitope regions, which are characterized by different selective pressures acting on the different genotypes^40^. Mutations in this protein may be attributable to escape from the host immune response during the prolonged circulation in a highly immune swine population^40^. According to the differences in size, charge, and hydrophobicity between amino acids, these differences may have major consequences on the secondary and tertiary structure of PCV2 capsid protein^43^. We summarized several differences between PCV2a and PCV2d-2 regarding the properties of amino acids substituted among four general antibody recognition regions, including F53I, A59K, A68N, T134N, S169G/R, R191G, K206I, K232N, and -234K. The results of the present study showed that residues 59, 63, 89, 130, 133, 206, and 210, which have been determined to be crucial for binding of the monoclonal antibodies (mAbs), were located on the surface of the capsid protein^44^. Based on this result, residues 59 and 206 have properties of amino acids that have been substituted, which may influence the vaccine-induced antibody binding abilities^44,45^. Moreover, the alanine (A) at position 59 in the PCV2a capsid protein is a crucial amino acid, because one neutralizing epitope was identified using a mAb, namely 8E4^45^. If residue 59 is switched from alanine (PCV2a) to arginine (PCV2b), the reactivity of mAb 8E4 is lost^45^. Arginine (PCV2b) and lysine (PCV2d) at position 59 have similar properties (basic amino acid), with pKa values of 12.48 and 10.54, respectively. Therefore, the consequence of residue 59 substitution from alanine (PCV2a) to lysine (PCV2d) may be consistent with PCV2b. In addition, residues 190, 191, 206 and 210 are crucial for PCV2 replication *in vitro*^29^. Our findings also showed that changes in residues 191 and 206 can affect the property at this site, and whether these change will affect PCV2d characteristics should be further investigated.

Residues 173–175 and 179 are crucial for antibody recognition^30^, and in our analysis, amino acids at these 4 positions in PCV2a, PCV2b, and PCV2d were conserved. This result was consistent with the finding that amino acids 169–180 of the capsid protein are highly conserved among all PCV2 genotypes^46^. Trible *et al*. suggested that this region of the capsid protein may serve as a decoy, diverting the humoral immune response away from a protective epitope^46^. Antibodies induced by the vaccination of virus-like particles (VLPs) primarily recognized the largest polypeptide, the capsid protein (amino acids 43–233), which is related to neutralization antibodies. In contrast, PDNS-affected pigs primarily produced the largest polypeptide as well as a small polypeptide, the capsid protein (amino acids 169–180), including an immunodominant domain comprising a short oligopeptide^46^. According to the X-ray crystal structure of the capsid protein (amino acids 40–233)^47^, the capsid protein (amino acids 169–180) region forms an external loop structure that protrudes from the outer surface of the PCV2 capsid protein subunit. The key antibody-binding residues F174, G175, and K179 were not visible in the VLP surface, except for Y173, which was visible on the surface of the PCV2 VLP and was located at the bottom of a cleft formed by the junction of three capsid protein monomers. However, these four residues, which are present on a single capsid protein subunit, are located in the middle of a connecting loop region and lie in the same plane^48^. In an experimental challenge study, the PCV2 VLP and the capsid protein (amino acids 43–233) monomer were used for vaccination. The results showed that the PCV2 VLP induced higher levels of anti-capsid protein (amino acids 43–233) antibodies than anti-capsid protein (amino acids 169–180). After PCV2 challenge, no PCV2 viremia was detected in the pigs vaccinated with PCV2 VLP. In contrast, immunization with the capsid protein (amino acids 43–233) monomer, which induced high levels of antibodies against the capsid protein (amino acids 43–233) as well as a highly elevated immune response to capsid protein (amino acids 169–180). Surprisingly, PCV2 viremia from animal immunized with the capsid protein monomer group was similar to that of unvaccinated PCV2-challenged group. These results suggested that the production of protective antibodies against the capsid protein (amino acids 43–233) is elicited from immune epitopes formed by the PCV2 VLP, whereas non-protective antibodies against the capsid protein (amino acids 169–180) are produced by exposure to the capsid protein monomer^46^. However, if the X-ray crystal structure of PCV2 capsid protein (amino acids 40–233) is correct, the high levels of anti-capsid protein (amino acids 169–180) should not be detected in PDNS-affected pigs, because the field PCV2 virus is an entire virus particle, and the key antibody binding residues F174, G175, and K179 should not be visible. Therefore, a better understanding of the capsid protein (amino acids 169–180) is still needed.

## Methods

### Ethics statement

This study did not involve any animal experiments. The Institutional Animal Care and Use Committee (IACUC) of the NPUST did not deem it necessary for this research group to obtain formal approval to conduct this study.

### Specimen selection and histopathological examination

Clinical samples were collected from the ADDC of NPUST from January 2016 to February 2017, and 1,092 pigs and 724 serum samples from central and southern Taiwan were received for pathological diagnosis and pathogenic molecular detection. According to previous studies, a PCV2 load higher than 10^7^ DNA copies/ml can be used to categorize pig as PCVAD^1,27^. The criteria for positive cases that were selected for ORF2 gene amplification in the present study were a PCV2 viral load of more than 10^8^ copies/mL for the serum sample or a quantitation cycle (Cq) value less than 22.8 in the homogenized tissue. Necropsy was performed and tissues were fixed in 10% non-buffered formalin for 24 hours for hematoxylin-eosin histopathological examination. Tissues (tonsil, hilar lymph node, lung, and spleen) and blood were routinely obtained from these pigs for pathogenic detection. PCV2 immunohistochemistry analysis was performed using the formalin-fixed lung and lymph node samples for typical PCV2 infection cases using the Novolink™ Polymer Detection System (Novocastra Laboratories, Newcastle upon Tyne, UK) and a 500-fold diluted rabbit polyclonal antibody against the PCV2 capsid protein (GeneTex, Inc., Irvine, CA, U.S.A.). PCV2 immunolabeling (brownish signal) was observed the cytoplasm of macrophage-like cells in lymphoid tissues.

### Sample preparation

Viral DNA was extracted from clinical specimens (either serum or homogenized tissues) using a MagNA Pure LC total nucleic acid isolation kit (Roche Diagnostics GmbH, Mannheim, Germany) operated on a MagNA Pure LC 2.0 automatic nucleic acid extraction instrument (Roche Diagnostics GmbH, Mannheim, Germany), according to the manufacturer’s protocol.

### Polymerase chain reaction (PCR) for PCV2 screening and complete ORF2 gene amplification

The primers PCV2-89F (5′-CGT-TGG-AAT-GGT-ACT-CCT-CAA-3′), PCV2-89R (5′-TGT-AGC-ATT-CTT-CCA-AAA-TAC-CAA-3′), and the Universal ProbeLibrary probe 33 (Roche Diagnostics GmbH, Mannheim, Germany), which targets the ORF1 gene, were used for quantitative real-time polymerase chain reaction to detect PCV2 in the clinical specimens. The entire ORF2 gene of PCV2 was PCR amplified by as described by Takahagi *et al*.^49^, after which the DNA fragments were purified (Geneaid Biotech, Ltd, Taipei, Taiwan) and the target nucleotide sequences were determined from both orientations using an auto sequencer (ABI 3730XL, Foster City, CA, U.S.A.)^25^.

### Sequence analysis

Two hundred fourteen complete PCV2 ORF2 sequences were analyzed, including 60 sequences from this study, sequences that were previously obtained from Taiwan from 2001 to 2017, and 46 PCV2 reference strains from NCBI (Table 1). The PCV2 ORF2 sequences were aligned using the Muscle method^50^. Translation of the nucleotide sequences and estimation of the genetic distance were implemented in MEGA version 7 (available online: http://www.megasoftware.net/)^51^. The phylogenetic trees were constructed using the maximum-likelihood (ML) method, and branch support was evaluated by bootstrap analysis based of 1,000 replicates for the ML tree.

### Phylodynamic and phylogeographic analysis

One hundred twenty-six PCV2 sequences from Taiwan were used for phylodynamic and phylogeographic analyses. For the phylogenetic molecular clock analysis, the PCV2 sequences were assessed using a temporal signal to identify data quality using TempEst^52^. The evolution rates of PCV2 sequences were estimated using the Bayesian Markov chain Monte Carlo (MCMC) method offered in BEAST v.1.10.2 (available online: http://beast.bio.ed.ac.uk/)^53^ along with the BEAGLE library^54^. SRD06 was used as the best-fit nucleotide substitution model because of its better resolution for coding regions for Bayesian analysis^55^. The demographic model, including constant size, exponential growth, Bayesian skyline, and Gaussian Markov random field (GMRF) skyride was used to estimate the evolutionary and population dynamics using strict, lognormal relaxed and exponential relaxed molecular clock models^56^. The MCMC chains were run for a sufficient time to achieve convergence (effective sample size > 200), and the best-fit demographic and clock model was estimated by marginal likelihood using path sampling and stepping-stone sampling^57^. An MCC tree was constructed using Tree Annotator v.1.10.2, with a 10% burn-in. The final phylogenetic trees were edited using Figtree v.1.4.2 (available online: http://tree.bio.ed.ac.uk/). The phylogeographic analysis was performed in BEAST v1.10.2^53^. A discrete trait substitution model was used for the symmetric substitution model with Bayesian stochastic search variable selection (BSSVS). Visualization of spatial phylogenetic and Bayes factor calculation was performed using SpreaD3^58^.

## Conclusions

In this study, we confirmed PCV2d-2 as the predominant PCV2 genotype in Taiwan. The introduction of PCV2 vaccination was indeed effective for PCV2 control in the field. However, the fast evolution rate of PCV2 may have led to the observed genotypic shift of this virus in Taiwan. The estimated nucleotide substitution rate of Taiwanese strains in the ORF2 region was 8.467 × 10^−4^ substitutions per site per year. TMRCA of PCV2, -2a, -2b, and -2d-2 were dated to 1957, 1970, 1992 and 2004, respectively. The genotypes PCV2a, -2b, and -2d were shown to have been present in Taiwan before introduction of a PCV2 vaccine. Further surveillance and monitoring of PCV2 evolution is necessary for disease control, and ORF2 residues 53, 59, 68, 134, 169, 191, 206, 232 and 234 had amino acids substitutions with different properties between PCV2a and PCV2d-2. These observations provide a foundation for researchers to consider new PCV2 vaccine design strategies and provide several directions for future study.
